# The first 1,000 days of life and early childhood caries: closing the global data gap

**DOI:** 10.3389/froh.2025.1701839

**Published:** 2025-11-19

**Authors:** Moréniké Oluwátóyìn Foláyan, Balgis Gaffar, Carlos Alberto Feldens, Robert J. Schroth, Francisco Ramos-Gomez, Jorma I. Virtanen, Hyewon Lee, Abiola Adeniyi, Maha El Tantawi

**Affiliations:** 1 Early Childhood Caries Advocacy Group, University of Manitoba, Winnipeg, MB, Canada; 2 AFRONE Network, Faculty of Dentistry, Alexandria University, Alexandria, Egypt; 3 Oral Health Initiative, Nigerian Institute of Medical Research, Lagos, Nigeria; 4 Department of Child Dental Health, Obafemi Awolowo University, Ile-Ife, Nigeria; 5 Department of Preventive Dental Sciences, College of Dentistry, Imam Abdulrahman bin Faisal University, Dammam, Saudi Arabia; 6 Department of Preventive and Social Dentistry, Faculty of Dentistry, Federal University of Rio Grande do Sul, Porto Alegre, Brazil; 7 Dr. Gerald Niznick College of Dentistry, University of Manitoba, Winnipeg, MB, Canada; 8 School of Dentistry, University of California, Los Angeles, Los Angeles, CA, United States; 9 Institute of Dentistry, University of Turku, Turku, Finland; 10 School of Dentistry, Dental Research Institute, Seoul National University, Seoul, Republic of Korea; 11 School of Public and Global Affairs, Fairleigh Dickinson University, Vancouver, BC, Canada; 12 Department of Pediatric Dentistry and Dental Public Health, Faculty of Dentistry, Alexandria University, Alexandria, Egypt; 13 Faculty of Dental Medicine, Alamein International University, Matrouh, Egypt

**Keywords:** early childhood caries, pregnancy, health policy, maternal and child health, global health, health inequities

## Abstract

The first 1,000 days of life represent a critical window for preventing Early Childhood Caries (ECC). However, a significant global data gap obscures the true scale of ECC within this critical period. This review aims to systematically examine the global availability of ECC data for children under 36 months, discuss age-specific prevalence trends, and synthesize evidence to highlight the implications of missing data. A comprehensive analysis of a global dataset reporting ECC prevalence across 193 United Nations member states (2007–2017) was conducted. Analysis of the data was organized by the World Health Organization Region. The analysis revealed a profound data gap: 73.6% of countries had no data for children under 36 months, and only 19.7% had current data. Where data existed, rates approach or exceed 50% in some countries (e.g., Egypt: 69.6%, Mongolia: 47.5%), indicating that ECC is often well-established in the first 1,000 days of life. Significant regional disparities were identified, with the highest burden in the European Region, the Eastern Mediterranean Region, and the Western Pacific Region. Even within regions, there are extreme disparities in prevalence between countries (e.g., Kuwait at 3.0% vs. Egypt at 69.6% in the Middle East; Finland at 0.3% vs. Kazakhstan at 45.0% in Europe). The scarcity of data and high prevalence rates highlight a public oral health problem in infancy. Closing this global data gap is an essential first step to mobilize resources and implement targeted, effective prevention strategies where we can have the greatest impact.

## 1 Introduction

The first 1,000 days of life represent a critical period for Early Childhood Caries (ECC) prevention. ECC is characterized by the demineralization of tooth structure that leads to both cavitated and non-cavitated lesions in the teeth of children younger than 72 months (1). It is the most common noncommunicable disease (NCD) worldwide in this age group, affecting 48% of preschool-aged children, and is the 10th most common childhood disease globally (2–4). The 2016 World Health Organization (WHO) Global consultation and the 2018 International Association of Paediatric Dentistry meeting, both held in Bangkok, have previously issued urgent calls to action to address this public health crisis (5, 6). Beyond its negative impact on quality of life, growth, and neurodevelopment (7, 8), ECC imposes a considerable economic burden on families and healthcare systems through direct treatment costs, including complex care under general anaesthesia, and indirect costs from missed learning time and caregivers' lost productivity (9–13).

The first 1,000 days of life, often called the “golden interval”, span the period from conception to a child's second birthday (14). Findings from birth cohort studies indicate that early-life experiences, encompassing the gestational period and the first two years of life, exert lasting effects on health outcomes (15, 16). In addition, the first 1,000 days represent a critical period for ECC prevention. The period includes the time for the development and eruption of primary teeth, oral microbiome acquisition and colonization, prenatal nutrition, and the initiation of oral hygiene routines. Exposure to harmful substances or stress during this period can increase the future risk of NCDs, including dental caries (17). Diet (18, 19) and dietary habits (20–25) established during this time play a pivotal role in shaping the oral microbiome and dental caries risk (26–29), with suggestive evidence that the acceleration in the incidence of dental caries during the first two years of life is never again observed throughout the lifespan (6). The first 1,000 days of life also represent a uniquely critical window for the foundational child development and a pivotal opportunity for parents to establish healthy behaviours that underpin long-term physical and emotional well-being (30). Maternal and paternal overall health status and maternal nutritional intake during pregnancy, parental avoidance of harmful substances like tobacco and alcohol, and minimization of exposure to environmental toxins such as air pollutants are intrinsically linked with the child's oral health (31). The mother's oral health status, dietary habits, knowledge of hygiene practices, and cultural practices are primary determinants of the child's susceptibility to ECC and the establishment of lifelong healthy infant dietary practices and oral hygiene routines (32). Therefore, interventions during these 1,000 days are essential for breaking the cycle of oral diseases and promoting holistic well-being from the very start of life.

ECC begins soon after the eruption of primary teeth, around six months of age, and the thinner enamel of primary teeth allows decay to progress rapidly in the absence of preventive measures (33, 34). Although interventions during this period, such as oral health education and the first dental visit by 12 months of age, are potentially effective, the reported impact remains modest, indicating the need to enhance such strategies across different contexts (26, 35). In addition, the common practice of reporting on children aged 0–5 years collectively in epidemiological studies and the lack of national policies for oral health screenings for those <36 months obscure insights into the earliest onset of ECC, limiting the development of targeted prevention policies and programs for high-risk periods among these five years, including the first 1,000 days of the child's life (36, 37). Taken together, these barriers represent critical issues that need to be addressed by the scientific community working in the field of child health to reduce the public health burden of ECC on children, families, and society. The purpose of this review is to systematically examine the global availability of ECC data for children under 36 months, highlight age-specific prevalence, and discuss the implications of the findings for future oral health-related policy and programmatic efforts focused on the first 1,000 days of life.

## 2 Data gaps for early childhood caries in the first 1,000 days of life

Data gaps for ECC in the first 1,000 days of life arise from challenges in representative sampling, accessing infants and toddlers, data quality, and measurement bias (38). Where there is data, there is inconsistency in the age bands and reporting, making it difficult to extract data for the first two years of life for children. There is also sparse data for children in this age group (37).

To quantify the global data gaps for ECC, a comprehensive analysis was undertaken using a dataset previously generated to report ECC prevalence across 193 United Nations member states between 2007 and 2017 (37). The selection of the time interval was to ensure that recent estimates, at the time of publication, were reported and to reduce the impact on the validity of shifting diagnostic criteria by time. The dataset distinguished between two key age groups: children under 36 months, representing the first 1,000 days of life, and children aged 36–71 months. Based on data availability, countries were classified into three categories as having: “no data”, reflecting a complete absence of ECC prevalence estimates; “data before 2007”, denoting outdated data collected more than ten years before the study was published; and “with data”, indicating the presence of ECC prevalence estimates from 2007 to 2017. For this last category, prevalence estimates were calculated at the country level by dividing the number of ECC-affected children by the total number of examined children across country-specific studies and rounding to one decimal place. The categories “no data” and “data before 2007” were combined to assess data gaps.

Table 1 shows that 142 countries (73.6%) had no data for children younger than 36 months, 13 (6.7%) had outdated data, and 38 (19.7%) had current data. This highlights a striking global scarcity of recent data on ECC during the critical first 1,000 days of life, when preventive interventions can have the greatest impact. The situation of data scarcity leaves the onset of ECC in its earliest stages largely hidden from public health planning and constitutes a serious public health problem, given that ECC can develop soon after tooth eruption, making early detection essential for timely prevention.

**Table 1:** Data availability for ECC prevalence in children under 36 months of age.

Data status	Number of countries	Percentage of countries
No data	142	73.6%
Data before 2007	13	6.7%
Data between 2007 and 2017	38	19.7%
Total	193	100%

The lack of data for children under 36 months is likely the direct consequence of significant methodological challenges inherent in studying this population. Data collection in infancy and toddlerhood faces unique logistical and ethical hurdles that are less pronounced in older age groups (38). Logistically, accessing a representative sample of infants is difficult; they are not enrolled in school systems (39), which provide sampling frames for older children. Infants and toddlers have low attendance rates at primary healthcare clinics where surveys might be conducted (40). Gaining cooperation from parents for clinical examinations can be challenging due to concerns about infant distress, time constraints, and a perception that dental assessments are unnecessary for young children (41). Ethically, obtaining reliable clinical data requires trained examiners to perform clinical examinations, which can be stressful for both the child and parent, raising concerns about the burden of non-therapeutic research (42). Furthermore, diagnostic criteria for non-cavitated lesions (white spot lesions), which are the earliest signs of ECC, can be difficult to apply consistently in young children who may have limited cooperation, leading to potential underestimation of the true disease prevalence (1). These challenges are compounded in low-resource and conflict-affected settings, where infrastructure is weak and survival needs take precedence over oral health surveillance (43, 44). These underscore the need for innovative, minimally invasive, and integrated approaches to oral health assessment in the first 1,000 days.

## 3 Prevalence of early childhood caries by region

Analysis of the El Tantawi et al. data set (37) revealed that there are geographic patterns in the missing data: Africa had the highest proportion of countries with no data at all, with 27 of 54 nations reporting no information for the age group. Conflict-affected areas such as Syria, Yemen, and South Sudan also stand out for their data voids. In contrast, outdated data, often more than 15 years old, occurs in parts of Latin America (e.g., Argentina and Guatemala) and Southeast Asia (e.g., the Philippines and Myanmar). This imbalance underscores the urgent need for oral health surveillance systems, especially in low-data regions, the standardization of ECC reporting across age groups, and updating outdated surveys to reflect current realities. Without closing these gaps, opportunities to address ECC in the first 1,000 days of life, when preventive care is most effective, will continue to be missed.

Further analysis of the dataset by the WHO regions (45) was conducted to identify the countries with the highest and lowest ECC prevalence rates. Table 2 presents the highest and lowest ECC prevalence rates specifically for children under 36 months by the WHO Region for those member states that reported data after 2007, highlighting the disparate burden of ECC within this critical period. Details of the country data per region are available in Supplementary File 1.

**Table 2:** Highest and lowest ECC prevalence in children under 36 months, by WHO region for the year 2007–2017.

Region	Country with the lowest prevalence in the region (%)	Country with the highest prevalence in the region (%)
African Region (AFR)	Nigeria (2.7)	Uganda (17.8)
Region of the Americas (AMR)	Columbia (19.2)	Canada (36.2)
South-East Asian Region (SEAR)	Sri Lanka and Thailand (24.5) respectively	India (38.9)
European Region (EUR)	Finland (0.3)	Kyrgyzstan (45.4)
Eastern Mediterranean Region (EMR)	Kuwait (3.0)	Egypt (69.6)
Western Pacific Region (WPR)	Japan (3.9)	Mongolia (47.5)

In several regions, the highest recorded prevalence for infants and toddlers approached or exceeded 50%, indicating that dental caries was established in nearly half of all children before their second birthday. The high prevalence in countries like Egypt (69.6%) and Mongolia (47.5%) indicates that for many children, the opportunity for primary prevention has already been missed by the preschool years. Therefore, the focus of public health efforts must target this age. Relying on interventions that begin at preschool age (3–5 years) is often too late. Targeted, context-specific strategies must be prioritized in high-prevalence regions like the European Region, Eastern Mediterranean Region, and Western Pacific Region.

**Figure 1:**
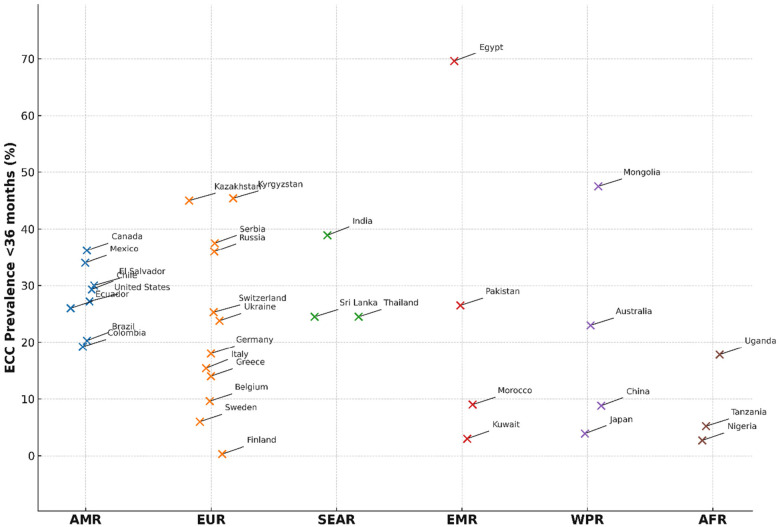
A plot of ECC prevalence in children under 36 months by country per the WHO region. Each dot represents the prevalence of a single country. Only the countries with the lowest and highest prevalence within each region are labeled. See Supplementary File 1 for details.

Figure 1 (with data presented in Supplementary File 1) shows that the highest average prevalence was observed in the EMR, where estimates exceeded 40% in several countries. In contrast, countries within the EUR had a lower prevalence, with median values below 20%. Still, the variability between countries was substantial. AFR showed wide dispersion, ranging from very low prevalence in Nigeria (2.7%) to over 30% in Namibia. AMR and SEAR exhibited moderate-to-high prevalence, with averages between 25% and 35%, while WPR showed relatively low prevalence overall but included pockets of higher burden (e.g., Mongolia at 47.5%). These findings highlight both cross-regional and within-region disparities, reflecting differences in health systems, preventive programs, and socioeconomic determinants. This means that by the time many children are old enough to attend preschool-based prevention programs, the disease is already well-established. Even in countries with moderate prevalence, the figures represent hundreds of thousands of infants who experience preventable diseases.

The high prevalence figures justify the urgency of interventions in this period, while the pervasive data gaps highlighted in Table 1 reveal a critical blind spot in global public health surveillance. Closing this data gap is an essential first step towards mobilizing resources and implementing targeted, effective prevention strategies when they are needed the most, at the very beginning of life. Without preventive measures before age three, the likelihood of significant ECC by age five becomes extremely high. This evidence reveals a “now or pay later” reality, where neglecting early intervention drastically increases the long-term burden of disease, complex treatment needs, and associated costs.

## 4 The dual crisis: data scarcity and high disease burden in infancy

The systematic analysis revealed a dual crisis: a profound lack of data for children under 36 months, and, where data exists, alarmingly high prevalence rates that signify ECC is often established at an early age. This reveals that for a significant number of children globally, the first 1,000 days of life are not just a period of risk, but a period of active disease. This high level of disease in early life fundamentally shifts the perspective on ECC prevention, framing it as a pressing public health emergency unfolding in infancy (46).

The lack of reliable data for younger children, when the foundation for oral health is laid, has significant implications. A child's caries risk is shaped by the family environment, particularly patterns of sugar consumption (30), with key drivers being socioeconomic and behavioural, especially lower maternal education and early sugar exposure (31, 47). Once established, ECC can destroy the whole tooth crown within just 6–12 months if left untreated (48). Without reliable prevalence estimates for under 36 months, the time of onset and true magnitude of ECC remain hidden, delaying targeted prevention efforts and misdirecting resources. The need to focus on ECC as a disease during the first 1,000 days of life is underscored by the European Academy of Paediatric Dentistry, which restricts its definition of ECC to those under 3 years of age (49).

The challenges in obtaining reliable oral health data for this age group are substantial, stemming from difficulties in sampling and accessing infants and the lack of national early childhood oral health policies (38, 50). However, these challenges are exponentially intensified and rooted in deeper structural inequities and systemic policy neglect. This foundational neglect is a root cause of both the pervasive data gaps and the stark disparities in ECC prevalence. Oral health surveillance requires dedicated resources, which are inherently costly and complex (51). Structural barriers, including weak healthcare systems, limited dental public health staffing, and poor infrastructure, severely limit data collection, particularly in rural, displaced, and refugee populations (51). These barriers are most acute in conflict zones and humanitarian settings, where oral health systems are chronically underpowered and access to care is disrupted (44, 52, 53).

A striking finding is the pronounced data void in Africa and conflict-affected regions, reflecting a complex intersection of systemic neglect, logistical barriers, and structural inequities with profound implications for child health (44, 54). This creates a significant reporting bias that obscures the full global picture. In addition, the high prevalence based on available data from these same regions indicates a high (true) disease burden. The data gap and high prevalence are two facets of the same crisis of neglect. Many under-resourced health systems, oral health remains a low priority, with scarce funding overwhelmingly directed toward infectious diseases and maternal-child survival, leaving oral health understaffed, poorly equipped, and disconnected from overall healthcare (55). This neglect is not confined to low-income nations; neglected populations of children in high-income countries like Australia, Canada, New Zealand, and the United States also carry ECC burdens well above the national average (56–58). Underpinning these practical barriers is a historical concentration of research agendas and funding in the Global North, creating a dependency that leaves the priorities of African and other vulnerable populations severely underrepresented.

The void of reliable ECC prevalence data for children under 36 months has major consequences. An absence of data may lead policymakers to assume ECC is not a problem, preventing the allocation of limited healthcare funding to its prevention and management. Resources are misallocated based on flawed assumptions or extrapolations from neighbouring countries, resulting in prevention strategies that are often grossly inadequate. This cycle perpetuates profound health inequities, as the children most vulnerable to ECC—those experiencing poverty, malnutrition, or displacement—are systematically uncounted and underserved. Globally, the absence of data from vast regions like Africa hinders the tracking of progress toward managing NCDs and universal health coverage, masking the true scale of the crisis.

To address this critical data gap, a concerted effort must be made to prioritize infant oral health surveillance globally. A key strategy involves implementing the “First dental visit by 12 months” and early establishment of the dental home concept, particularly in data-poor regions, to facilitate early detection and establish baseline data (48, 59, 60). Another pragmatic approach involves integrating oral health modules into existing maternal and child health care systems in the context of primary health care, and integrating oral health assessment into large-scale population health surveillance surveys, such as Demographic and Health Surveys (61) or Multiple Indicator Cluster Surveys (62). National surveys like the NHANES in the United States, which provide robust, nationally representative data for children aged 1–5 years, serve as a valuable model (63). Simultaneously, building local research capacity through equitable partnerships and sustainable funding models is critical to ensure ownership and long-term sustainability. Innovative methods, such as task-shifting to community health workers, could help reach the most inaccessible populations. Ultimately, strong advocacy is needed to push oral health higher on the global child health agenda. Addressing this data gap is an essential first step toward health justice (57), empowering countries to combat a preventable disease that continues to harm the youngest and most vulnerable.

Another striking finding is that where data is available, the prevalence figures are very high. The high prevalence by age three in many countries means that public health strategies focusing on preschool-aged children (3–5 years) often initiate prevention too late, after the disease has already been established. The common practice of grouping 0–5-year-olds in epidemiological studies obscures this crucial distinction. Furthermore, the extreme disparities in prevalence between neighboring countries and within regions (e.g., Kuwait at 3.0% vs. Egypt at 69.6%; Finland at 0.3% vs. Kazakhstan at 45.0%) highlight that ECC is not an inevitable outcome of early childhood. Instead, it is a preventable disease whose trajectory is determined by a complex interplay of social, economic, behavioral, and policy factors. These disparities point to the importance of context-specific, culturally appropriate interventions rather than a one-size-fits-all global approach.

The disparities in ECC prevalence between countries may reflect the profound impact of a cascade of influences, where a country's macro-level profile directly dictates micro-level realities for children and families. A nation's overall socio-economic situation establishes foundational inequality; in high-income countries, comprehensive public health policies and water fluoridation provide a protective macro-environment, whereas in lower-income nations, poverty and inadequate infrastructure create a high-risk baseline. This macro-level disparity manifests in a family's ability to afford taking caries prevention approaches, including eating nutritious food and buying oral hygiene products. Furthermore, the population's general educational attainment influences parental health literacy, determining a caregiver's knowledge of feeding practices and preventive care at the individual child's level. Finally, the structure and accessibility of a country's health services determine whether a family has access to affordable, preventive dental care or relies on emergency treatment, cementing the cycle of disease. Consequently, the disparities in ECC prevalence between countries are not merely a collection of individual choices, but rather the direct result of how economic, educational, and health-based macro-level systems intersect to create vastly different micro-level lived experiences and health outcomes.

The implications of these findings are important. First, there is an urgent need to prioritize and fund standardized surveillance systems specifically designed to capture ECC data in the under 36 months globally. This is the essential first step to unmasking the true scale of the problem and enabling targeted resource allocation. Second, the focus of preventive efforts must be directed to an earlier age, integrating oral health into maternal and child health platforms during pregnancy and the first two years of life. Interventions must engage expectant and new parents, emphasizing the mother's role as the primary determinant of the child's early oral health environment through her own oral health, nutritional choices, and hygiene practices.

## 5 Study strengths and limitations

This study makes a valuable contribution to the field of global oral health by highlighting the scarcity of data on ECC in children under 36 months. Focusing on this early age is important, as it represents a key period for potential preventive measures. The analysis of a dataset covering 193 United Nations member states gives the study a broad, global perspective, allowing for useful comparisons between different regions. The findings illustrate notable differences in ECC rates both between and within WHO regions, helping to emphasize that ECC is influenced by local conditions. The study also offers practical suggestions, such as incorporating oral health into existing child health surveys and encouraging early dental visits, which can help guide policy and advocacy efforts.

The analysis, however, relies on a single comprehensive dataset (37). While this provides a standardized overview, it may not capture all data from national health reports or smaller cohort studies that were not identified in the original systematic review. Furthermore, the prevalence estimates within this dataset were standardized at the country level by calculating a mean estimate using available studies for that country, rounding to one decimal place. This approach provides a general estimate but may mask subnational variations and does not fully eliminate the heterogeneity inherent in combining data from multiple independent studies. In addition, the variability in applying the diagnostic criteria for caries (i.e., whether considering cavitated lesions or both non-cavitated and cavitated lesions) in the absence of calibration across studies, the inconsistencies in the age bands reported, and the aggregation of data from studies with different sampling methodologies present challenges for direct cross-country comparison. Therefore, while the presented figures highlight alarming prevalence rates and critical data gaps, they should be interpreted with an understanding of the underlying methodological heterogeneity. Future global surveillance efforts would benefit from stricter standardization of age-specific reporting and diagnostic protocols for the under 36 months population.

## 6 Conclusion

The analysis underscores a critical lack of data, and, where data is available, the high prevalence rates indicate that ECC is often established very early in life. Neglecting intervention during the first 1,000 days drastically increases the burden of ECC and its adverse consequences for children and their families, necessitating more complex, costly, and traumatic treatments under general anesthesia, while also incurring significant indirect costs due to caregivers' lost productivity. Conversely, investing in early preventive strategies promises substantial returns by reducing suffering, mitigating health inequities, and lowering economic costs for families and healthcare systems. The data gap can be closed by integrating oral health assessments into existing maternal and child health platforms, such as antenatal care and well-baby visits, and into large-scale population surveys. Standardizing ECC reporting globally by adopting consistent age bands, particularly 0–36 months, and uniform diagnostic criteria that include non-cavitated lesions, is important. Furthermore, the importance of promoting the “first dental visit by 12 months” as a cornerstone for both early prevention and improved data collection, especially in regions where information is scarce, is an important strategic action for prompt diagnosis and instituting preventive actions. Finally, closing the data gap and acting decisively on the available evidence is not just a scientific imperative but also a moral action that empowers countries to combat a preventable disease that disproportionately harms their youngest and most vulnerable children.

## Data Availability

The original contributions presented in the study are included in the article/Supplementary Material, further inquiries can be directed to the corresponding author.
