# Supplementary material for: The first 1,000 days of life and early childhood caries: closing the global data gap
Source: Front Oral Health. 2025 Nov 19;6:1701839. doi: 10.3389/froh.2025.1701839 (PMC12672514; doi:10.3389/froh.2025.1701839)
Supplement: Supplementary file 1 [file Table1.docx]

Supplemental File 1: **Summary of Early Childhood Caries by WHO Region**

**Region of the Americas (AMR)**

| Country | ECC Prevalence <36m (%) |
| --- | --- |
| Brazil | 20.3 |
| Canada | 36.2 |
| Chile | 29.3 |
| Colombia | 19.2 |
| Ecuador | 26.0 |
| El Salvador | 30.0 |
| Mexico | 34.0 |
| United States | 27.2 |
|  |  |

**European Region (EUR)**

| Country | ECC Prevalence <36m (%) |
| --- | --- |
| Belgium | 9.6 |
| Finland | 0.3 |
| Germany | 18.0 |
| Greece | 14.0 |
| Italy | 15.4 |
| Kazakhstan | 45.0 |
| Kyrgyzstan | 45.4 |
| Russia | 36.0 |
| Serbia | 37.5 |
| Sweden | 6.0 |
| Switzerland | 25.3 |
| Ukraine | 23.8 |
|  |  |

**South-East Asian Region (SEAR)**

| Country | ECC Prevalence <36m (%) |
| --- | --- |
| India | 38.9 |
| Sri Lanka | 24.5 |
| Thailand | 24.5 |

**Eastern Mediterranean Region (EMR)**

| Country | ECC Prevalence <36m (%) |
| --- | --- |
| Egypt | 69.6 |
| Kuwait | 3.0 |
| Morocco | 9.0 |
| Pakistan | 26.5 |

**Western Pacific Region (WPR)**

| Country | ECC Prevalence <36m (%) |
| --- | --- |
| Australia | 23.0 |
| China | 8.8 |
| Japan | 3.9 |
| Mongolia | 47.5 |

**African Region (AFR)**

| Country | ECC Prevalence <36m (%) |
| --- | --- |
| Nigeria | 2.7 |
| Tanzania | 5.2 |
| Uganda | 17.8 |
